# Replicability. Politics and Poetics of Accountability, Validation and Legitimation

**DOI:** 10.3389/fpsyg.2020.608451

**Published:** 2021-01-15

**Authors:** Giampietro Gobo

**Affiliations:** Department of Philosophy, University of Milan, Milan, Italy

**Keywords:** replicability, reliability, validity, politics of validation, credibility, repeatability, reproducibility, findings legitimation

## Abstract

Replicability is a term that not only comes with different meanings in the literature of many domains but is often associated or confused with other terms such as ‘reproducibility,’ ‘repeatability,’ ‘reliability,’ ‘validity,’ and so on. To add to the confusion, it can even be used differently across diverse disciplines. Though all named concepts are important, what makes them barely advantageous is that they do not cover some peculiar aspects of the replicability and validation processes, i.e., appropriateness of conceptualization; trustworthiness of operational definition and operational acts; accuracy of researcher’s description, categorization and/or measurement; successfulness of observational (or field) relation. Moreover, in social sciences and organization studies, the concept of validity of data is highly questionable due to the quite frequent shortage of real statuses of the observed objects. The present paper aims to challenge the received view on the concept of ‘replicability,’ by proposing a “situational approach” based on the idea that replicability works under certain organizational and socio-technic conditions, and that it is heavily influenced by the way that different stakeholders (scientists, technicians, participants artifacts, and technologies) respond to them. Consequently, it is important to understand how and why replicability works in different contexts. Its main purpose, without denying the importance of current conventional perspectives on replicability and its siblings, is to widen and change them to include an organizational setting and a reflexive epistemology. This implies the pursuit of a third way of replicability, between the postmodernist negation of its possibility and its opposite, i.e., a naïve naturalism. A way asserting that replicability is a jigsaw puzzle or a mosaic, constituted by discursive practices (poetics) and organizational achievements guiding the politics of accountability, validation and legitimation. The domain here considered pertains to the social and organizational sciences. However, though going beyond the aim of this essay, many issues could be reframed and adapted to medical, natural and physical sciences, as some of the following examples can show.

## Replicability: An Ambiguous Term

Replicability is an ambiguous term. In fact, in the literature of many domains, it has assumed different meanings, covered different concepts, and even intersected other terms thus often being associated or confused with ‘reproducibility,’ ‘repeatability,’ ‘reliability,’ and ‘validity,’ among some best-known terms. Moreover, beyond organization studies, these terms are used differently across diverse disciplines.

For example, reviewing the use of the terms ‘repeatability,’ ‘reproducibility,’ and ‘replicability,’ Plesser (2017) and Barba (2018) outlined different usage, posing them in a hierarchical order from down (lower validity of the results) to top (higher validity of the findings):

• *repeatability* (same team, same experimental setup): the measurement can be obtained with stated precision by the same team, using the same measurement procedure, the same measuring system, under the same operating conditions, in the same location on multiple trials. For computational experiments, this means that researchers can reliably repeat their own computation.
• *replicability* has at least two meanings, depending on the disciplines:
  (a) instances in which a researcher collects new data to arrive at the same scientific findings as a previous study (econometry, epidemiology, clinical studies, internal medicine, physiology, computational biology, biomedical research, and statistics);
  (b) a different team, same experimental setup: an independent group (using the same measurement procedure, the same measuring system, under the same operating conditions, in the same or a different location on multiple trials) arriving at the same results using the original author’s artifacts (microbiology, immunology, and computer science);
• *reproducibility* has at least two meanings, depending on the disciplines:
  (a) instances in which the original researcher’s data and computer codes are used to regenerate the results (econometry, epidemiology, clinical studies, internal medicine, physiology, computational biology, biomedical research, and statistics);
  (b) a different team, different experimental setup: independent researchers with a different measuring system, in a different location on multiple trials, obtaining the same results using their own data and methods (microbiology, immunology, and computer science).

In addition, Barba (2018) states that in political science and economics (as well as in organization studies, I suspect) the three terms are used with no distinction between them.

In order to unravel this skein, it is useful to go back and explore the epistemological ground of these concepts, starting from the criticism to the concept of replicability and the epistemological problem of the “regression of the experimenter.” They will allow us to set (on a different epistemological ground) the issue of replicability.

## Replicability: A Weak Concept

More than a century ago, Pierre Duhem, a French physicist and mathematician, proposed a thesis, known as the ‘Quine-Duhem’s thesis’ after being taken up by the American philosopher and logic Willard Quine, stating that:

“The physicist can never submit to the control of his experience an isolated hypothesis, but only a whole set of hypotheses. When experience is in disagreement with his predictions, it teaches him that at least one of the hypotheses, which constitutes the whole, is unacceptable and must be modified, but it does not indicate which one must be changed… [Therefore] a physics experiment can never condemn an isolated hypothesis, but only a theoretical whole” (Duhem, 1906: 211).

Consequently, according to Duhem, in physics an *experimentum crucis* is not possible, because no thesis can be compared in isolation with the experience, as it should be to allow the *experimentum crucis*: no theory compares itself with the results of an experiment. In fact, each theory subjected to experimental verification always comes together with many auxiliary assumptions, some of them even implicit, such as the ones concerning the functioning of the experimental apparatus. Since numerous hypotheses in mutual relation are contained within every theoretical model. E.g., the Darwinian evolution or the theory of relativity, the possible experimental falsification of the model does not indicate which of the many hypotheses is wrong, as exemplified in the following words of the sociologist of science Harry Collins (2004, 2017).

**Replicability and Gravitational Waves**

In the 1960s, the American physicist Joseph Weber built the first prototype of a gravitational wave detector, a physical phenomenon whose existence had been predicted more than half a century earlier by Albert Einstein’s theory of relativity. According to the *field equation* – the fundamental equation of general relativity – the sudden change of mass of a body would produce ripples in space-time. According to Einstein, these undulatory perturbations had to be extremely widespread in the universe, but at the same time very difficult to detect because extraordinarily weak.

The instrument built by Weber, in a basement of his department at the University of Maryland, consisted of aluminum cylinders 2 meters long and one meter in diameter. When a gravitational wave passed, these cylinders would register its transit thanks to a variation of 10^–12^ millimeters (proportionally, it is as if the distance from the sun to the nearest star varied by the diameter of a human hair – just to give an idea). Weber had invested years of his career in building these detectors, spending months in the laboratory and learning about them in every detail. In the early seventies, Weber published the first results and said he had recorded a first high flow of gravitational waves.

Weber’s publication was immediately received with considerable skepticism. The results in fact contained a fundamental contradiction: although the confirmation of the existence of gravitational waves was a proof in support of Einstein’s theory, the intensity of the waves that Weber had recorded was instead much higher than expected. This suggested, again according to the theory of relativity, that the universe should have an extremely short life; and the community of physicists considered it very unlikely. Here the first had the theory of relativity been refuted or confirmed by Weber’s observations? According to Weber the theory of relativity had been confirmed, but it required some adjustments in order not to provide very unlikely predictions about the life of the universe. According to the critics of the experiment, on the contrary, the intensity of gravitational waves recorded was simply incompatible with relativity and therefore Weber’s observations had to be necessarily wrong.

To disprove Weber, half a dozen research groups over the course of a few years built detectors similar to those of the University of Maryland, with the intention of repeating the experiment and invalidating it. Therefore, it was: no detector had reproduced the same results obtained by Weber.

Moreover, here is the second puzzle: did not Weber’s critics, who had denied his observations to rehabilitate relativity, contradict relativity itself with the failure to detect gravitational waves? In addition, how to decide who, between Weber and his rivals, had prepared the experiment in the most appropriate way for the study of such an elusive phenomenon? Of course, negative results were repeated several times, but this did not necessarily mean that gravitational waves did not exist. On the contrary, the lack of replicability could be due to the inexperience of Weber’s contenders, who could not boast his experience in designing such sophisticated instruments. While many scientists agreed that Weber’s claims were not credible, the reasons for their skepticism were very different, and sometimes contradictory. In particular, IBM physicist Richard Garwin had strongly resisted Weber’s conclusions on principle; and only to make his attack even more convincing he had made measurements that had dealt with a decisive blow to Weber’s hypothesis.

The dispute lasted throughout a decade and Weber committed himself to demonstrate how each rival experiment was somehow a victim of methodological fallacies. Therefore, he tried, in turn, to discredit those who had attacked him.

Starting from the beginning of the 1980s, the majority of the scientific community lost interest in the research of gravitational waves. This phenomenon, in fact, not only proved difficult to identify, but also slowed down the careers of many brilliant physicists who had dedicated themselves to it, because of shortage of funds and the general skepticism due to the continuous controversies within the community.

It was only in the late 1990s that the hunt for gravitational waves was relaunched with the appearance of a new generation of interferometric antennas, whose design retained some of the key elements of the instruments designed by Weber. Finally, the gravitational waves were then detected: on February 1, 2016, a joint press conference of the two research groups that had collaborated on the project [LIGO (Laser Interferometer Gravitational-waves Observatory) and Virgo (from the name of a cluster of galaxies in the constellation Virgo)], announced that in September 2015, they had measured the gravitational waves resulting from the collision of two black holes about 1.3 billion light years away from the solar system. Ironically, traces of one of the rarest events in the universe hit the new detectors only 2 weeks after they were activated, ending in a very short time a hunt that had lasted for a century and was expected to last for many more decades.

After the confirmation of the existence of gravitational waves, that won the Nobel Prize in 2017 to Kip Thorne, Barry Barish and Rainer Weiss (meanwhile Weber had died in 2000), nowadays, within the scientific community, there is an almost general consensus. Even if the consensus may not be absolute, it has now reached such a level that it would be very difficult for a skeptical physicist (on the interpretations of the data collected by Ligo and Virgo) to be considered credible enough to publish a scientific article in a prestigious journal.

In a nutshell, the results provided by the experiment that Weber had designed on the Einstein’s theory of relativity were only partially consistent with it. In fact, while the existence of gravitational waves seemed to provide an empirical confirmation of relativity, the excess of gravitational waves compared to the above-mentioned ones posed many questions. Not even the construction, by six other research groups, of as many instruments to check the reliability of Weber’s measurements solved the puzzle. In fact, in Weber’s opinion, the measurements made by his colleagues did not represent a defeat of his hypothesis as their instruments were not reliable. Gravitational wave detectors are actually very delicate apparatuses, and none of the other physicists had the same skills as Weber to build them in a very short time. Thus, he conceived that these new instruments did not confirm the existence of gravitational waves just because of the inexperience of his colleagues. Scientists were then confronted with questions such as what was not working and which of the experiments had to be rejected. If the scientists had followed the Popperian falsificationism (the single case that can refute a theory), they would have had to reject the theory of relativity, until then considered correct, because they disagreed with the measurement of the intensity of gravitational waves. But, it was not so, as noted above (However, as noted above, it did not happen).

Duhem’s position was expanded by Quine (1951) who gave it a much broader field of application than physics, extending it not only to scientific methodology but to all our knowledge, and especially to our language. In fact, while Duhem’s epistemological holism questions the possibility of a conclusive empirical falsification of single scientific propositions, Quine’s semantic holism goes further and believes that all our theoretical statements, not only the scientific ones, are devoid of separate meaning. In other terms, one cannot speak of the meaning of a statement considered individually, because the meaning is not discrete, but is continuum and rather “scattered” in the whole language. For Quine, only language, as a unitary whole, is properly significant.

## Experimenter’s Regress

These two olisms, collected under the so-called Quine-Duhem thesis, received further support by Harry Collins (1981), with his concept of “experimenter’s regress.” According to it, when the results of an experiment differ from the theory, scientific practice never follows only methodological criteria in deciding whether the experiment or the theory is wrong. The decision is made also through a complex set of social negotiations, based on criteria that go beyond logics and methodology, e.g., scientists’ reputation, rhetoric, network alliances, usefulness of the discovery, etc.

Even in the case of gravitational waves, we can clearly see as the empirical evidence is not direct and self-standing: every experiment (both Weber’s and his colleagues’) was based on a wide range of implicit premises and assumptions. Whether the experiments were always and unequivocally considered as a confirmation or a denial of a hypothesis (that derives from a theory), both Weber’s experiment and those of his colleagues would have questioned the theory of relativity: in the first case for the incompatibility with the estimation of the age of the universe; in the second case for the absence of gravitational waves. Facing this dilemma, a large part of the community preferred to question the credibility of Weber’s experiment (discrediting his instruments) and “save” relativity.

Collins calls this phenomenon “experimenter’s regress”: the repetition of an experiment with a different instrument serves as a confirmation test only if we are sure of the reliability of the second detector, and not vice versa; that is, it is not the repeatability of the results of an experiment that allows us to evaluate the reliability of the instrument. In fact, if we repeatedly measure the temperature with a broken thermometer, the results may also be stable and reproducible; however, not true:

‘an experiment is a valid test for a theory if it is constructed to give a correct result. However, how do we know if the result is actually correct? To get a correct result, you need to use a reliable instrument. But again, how do we judge the reliability of an instrument, if not by the correctness of the data provided?’ (Collins and Pinch, 1993: 98).

But this is tautological reasoning. Yet scientists, sooner or later, manage to break this tautological circularity by reaching a consensus. This type of conflict can therefore be overcome, but not based solely on an allegedly objective scientific logic. In fact, the achievement of some kind of consensus across the community (though almost never unanimous) can only be rooted in the multitude of social forces that come into play in a scientific controversy. Instead of making use of formal criteria, each statement is evaluated in the light of a series of social criteria: the reputation of the scientists who formulated it; their experience in that specific field; their confidence in the tools used to obtain evidence; the possibility of obtaining funds to continue research in one direction rather than another; the potential usefulness of the discovery, and so on.

According to Collins, the experimenter’s regress concerns all experiments. However, when there is already consensus on what result to expect, the problem does not arise explicitly. Unlike, when the object of study is new or controversial – and therefore there is no consensus of any kind about the nature of the measured phenomenon, which result is more plausible to obtain or which criteria to use to validate the collected observations – then the experimenter’s regress becomes more explicit.

However, this constructivist position does not aim to compete epistemological criteria (in this case traditional rational logics) against social forces (such as reputation, rhetoric, convincing style of writing, cultural assumptions, etc.) because when we analyze case-by-case the negotiations leading to the resolution of this type of dispute, there is no distinction between the two levels. What will seem more “logical” to the community, it will be determined by the credibility of the social actors involved, by how much the scientific community has already invested in a theoretical position or in an experiment, by the rhetorical capacity of scientists to bring colleagues and public opinion on their side.

## Moving to a Situational and Organizational Approach

If the concept of replicability (and its siblings) is very problematic in hard sciences, it is even more so in organization studies, and social sciences in general. In fact, in these areas we are dealing with animated beings, capable of producing meanings and behaviors that can change between one observation and another; as had already been stated in the “debate on method” (*Methodenstreit*), developed in Germany at the end of the XIX century.

However, the conclusions and influence of the *Methodenstreit* have reached and penetrated only some organizational and sociological approaches. Hence, starting at least from the 1910s (with the making of the Chicago School), no less than two tendencies coexist, represented by the contrast between quantitative versus qualitative methods, which have in their background the adherence or not to the conclusions of the *Methodenstreit*.

The former, roughly defined as “positivist,” imported the validation criteria of results from the natural sciences and tried to adapt them to the logic of inquiry of the social sciences. Among others, Goode and Hatt (1952); Cronbach and Meehl (1955), Bailey (1978); Carmines and Zeller (1979), Zeller and Carmines (1980), Fowler (1988), Babbie (2008) represent good examples. Within this perspective, the problem of legitimating research findings has been usually approached via two main concepts: *reliability* (of the measurement instrument) and *validity* (of data). These concepts originated in the hard sciences (in particular astronomy and psychometry), aiming to prevent both systematic and accidental errors.

The latter, defined (similarly loosely) “interpretativist,” rejected this tendency as a positivist worry, although with few authoritative exceptions as Cicourel (1964); Galtung (1967), Glaser and Strauss (1967); Denzin (1978).

However, after long resistance, in the early 1980s the terms ‘reliability’ and ‘validity’ were introduced into qualitative research as well, with substantial adaptations (i.e., different meanings) by methodologists like Kirk and Miller (1986); Fielding and Fielding (1986), Hammersley (1987, 1990, 1992); Silverman (1993, 2000). This change was important because it aided qualitative research to rebut criticism concerning its romanticism and excessive subjectivity, and to establish standards for the credibility and consistency of its findings. However, this opening-up has been (internally) attacked as a ‘mere positivist concern’ by the feminist and postmodern critiques which diagnose a ‘legitimation crisis’ (Denzin and Lincoln, 2000: 17), i.e., the qualitative research cannot be evaluated with such conventional criteria.

Anyway, from the 1990s these two terms have become detached from the label of positivism and imported into the qualitative paradigm. In addition, new terms and concepts have been proposed in order to deal differently with this matter:

• *completeness* of descriptions (Miles and Huberman, 1994: 279);
• *saturation* of categories (Glaser and Strauss, 1967);
• *authenticity*, as certification of the researcher’s presence in the setting;
• *ecological validity* (Cicourel, 1996);
• *consistency* (Hammersley, 1992: 67);
• *credibility*, as a bridge between a researcher’s interpretation and ‘reality’ (Agar, 1986; Hammersley, 1990: 57 and 61; Miles and Huberman, 1994: 279);
• *plausibility*, as the coherence between researcher’s findings and theories accepted by the majority of the scientific community.

The whole debate has been well summed up by the expressions ‘the quality of qualitative research’ (Seale, 1999) and ‘the credibility of qualitative research’ (Silverman, 2006: 271ff), where quality refers to the transparency of the whole research process, and credibility pertains to the validation of findings and results.

Along with new proposals for:

ensuring a more rigorous research *design* (Marshall and Rossman, 1989; LeCompte et al., 1993; Mason, 1996; Maxwell, 1996),

• *sampling* appropriately (Corsaro, 1985; Strauss and Corbin, 1990; Becker, 1998),
• collecting *fieldnotes* systematically (Schatzman and Strauss, 1973; Emerson et al., 1995),
• constructing *models* with ethnographic data (Corsaro and Heise, 1990),
• *communicating* the results (Wolcott, 1990; Marx, 1997).

Bryman (1988, 1989) introduced this methodological heritage into organization studies.

Despite these conceptual improvements, what is often left by the epistemological and methodological debates about the nature of data (as treated by positivism, critical realism, constructionism, relativism and postmodernism) is a radical organizational approach, in which replicability works under certain organizational and socio-technic conditions, and it is heavily influenced by the way that different stakeholders (scientists, technicians, participants, artifacts and technologies) respond to them. This approach leads to a “situational” (or contextual) vision of replicability, considered case-by-case, study-by-study, method by method. For example, if the aim of the concepts of reliability and validity is to prevent both systematic and accidental errors, the ethnographic method already has an advantage over other methods, because some of these errors (due to the circular nature of the ethnographic research) can be corrected step-by-step as the research proceeds. In other words, an abstract and philosophical treatment for dealing with the issues of quality and credibility could be misleading.

In conclusion, reliability and validity (at their inception) were certainly important concepts because they raised relevant questions, overlooked by a “romantic” drift (Silverman, 1989) in organization studies and social science, with respect to the credibility of the research findings. However, the theoretical and technic solutions emerged from these two concepts are not adequate and turned out to be of limited usefulness in social and organizational research, because they neglect a number of important issues.

Particularly, reliability does not cover at least five aspects of the validation process such as:

• appropriateness of conceptualization (see section “Appropriateness of Conceptualization”),
• trustworthiness of operational definition and operational acts (see section “Trustworthiness of Operational Definition and Operational Acts”),
• accuracy of researcher’s description, categorization and/or measurement (see section “Researcher’s Accuracy”),
• fidelity of data and of interpretations (see section “The Fidelity of Data and of Interpretations”), and
• successfulness of observational (or field) relation (see section “The Observational Relation”).

In addition, the concept of validity of data is highly questionable in social and organizational sciences for the shortage of real statuses of observed objects.

For these reasons, the problem of replicability and validation of research findings should be rethought and reframed starting from the above listed aspects. The validation could be re-considered as tiles of a mosaic or pieces of a jigsaw puzzle, which should be detected, argued and inserted. This is due to the compositional nature of the replicability, which is built on different aspects simultaneously concurring to determine the goodness of the findings of a research. However, being aware that not always all the tiles can be available because (given the human nature of both the participants in the research and the researchers themselves) there will always remain aspects that can escape the eye of the researcher. However, taking these five aspects seriously may lead researchers to a certain degree of confidence about the validity of their findings.

It is time now to review these aspects, starting from the epistemological, methodological and technical weaknesses of the concept of reliability.

## Reliability of the Gathering Instrument

In conventional literature, reliability refers to the precision of the *instrument* (or method) of measurement. When methodology textbooks introduce the concept of ‘reliability,’ they usually exemplify it with the thermometer, or with weighing scales: if we repeatedly weigh the same object and obtain similar results, and if the values are the same when a second set of scales is used, we may say that the first scales (and reflexively or tautologically, also the second) are reliable. Therefore, reliability concerns our confidence in the consistency of a gathering instrument – its capacity to replicate results.^1^

Hence, the presence of a second instrument becomes essential. In fact, the reliability of one instrument only does not automatically lead to the validity of the measure, because the instrument itself may be faulty but nonetheless perfectly reliable – as the quantitative methodologists Edward G. Carmines and Richard A. Zeller exemplify:

‘let us assume that a particular yardstick does not equal 36 inches; instead, the yardstick is 40 inches long. Thus, every time this yardstick is used to determine the height of a person (or object), it systematically underestimates height by 4 inches for every 36 inches (…) However, this error of 4 inches per yard will *not affect* the reliability of the yardstick since it does not lead to inconsistent results on repeated measurements (…) In short, this particular yardstick will provide a quite reliable but totally invalid indication of height’ (1979, 13).

### What Is an Instrument? What Is Reliability?

The following example entails that at least two identical instruments are needed to evaluate the reliability of an instrument. However, what is an instrument? Is a questionnaire or a focus group an instrument? Similarly, can the questions inserted in these methods be considered as instruments? In addition, what about the response format of the questions? It may be the case that the questionnaire is suitable for gathering some information, but a particular question or the format that collects the answers is not. For example, Peterson (1984) conducted an experiment to observe the effects of various operationalizations of the ‘age’ variable. In a telephone survey of a probabilistic sample of 2083 American voters (only 63.5% of whom agreed to the interview), he asked their age in four different ways (one closed-ended and three open-ended questions), thus creating four different sub -samples (See Table 1).

**TABLE 1:** Peterson’s experiment on diverse operationalizations of the variable ‘age.’

**Question**	**Number of interviewees who answered the question**	**% of interviewees who lied (about their true age)**	**refusals**
How old are you?	299	1.3%	9.7% (32)
What is your age?	332	2.7%	3.2% (11)
In what year were you born?	282	2.8%	5.7% (17)
Are you _____? (listing the age groups)	347	4.9%	1.1% (4)
Total: 1324 interviewees	1260		

The experiment shows how sensitive a factual question like age can be. It also shows how the first operationalization obtains the most truthful results (but also the highest rate of refusals), while the closed-ended question is the least reliable, yielding the most false answers (but also the lowest refusal rate). Quite a dilemma, because each operationalization has its advantages and disadvantages.

Beyond this example, even the presence of two identical instruments (e.g., two questionnaires) is not always sufficient and does not solve the check of reliability. Let us consider some different situations:

• suppose we ask the same questions (about an opinion or a behavior), at different times (i.e., we made two observations/measurements), to the same participant or informant and we have always received the same reply;
• suppose we ask the same questions by two different interviewers to the same participant and we have always collected the same reply;

Can we say that the finding is necessarily true (i.e., valid) because it is consistent and replicable? No, because the participant or informant may have lied on both occasions^2^ or the question may have been formulated inadequately or the questionnaire is not the appropriate method to elicit such opinion or get the information about that behavior. Herein resides the entire paradox of reliability: the data may be erroneous but the method itself is reliable (the “quixotic reliability,” as defined by Kirk and Miller, 1986).

In addition, from epistemological point of view, the previous hypothetical reasoning has been based on the tacit assumption that the information (or the status on a property) collected does not change between the times of the two measurements. However, whereas Mr. Brown’s weight or height is relatively stable over time, his opinions may (and sometimes do) change quite rapidly. Faced with this change, there are four different possibilities:

(a) the questionnaires have correctly detected the change, because the participant has actually changed opinion between one administration (observation) and another;
(b) the first answer was true and the second false;
(c) the participant had lied the first time and now instead he has told the truth;
(d) the participant has always lied, in both administration.

When investigating an opinion (and not a fact as age, sex, residence, etc.), how can we know if the tool we are using is reliable or not? It might be impossible to find out. In the case of the measurement of an opinion or an attitude, the reliability of an instrument undergoes the circularity described as the problem of the experimenter’s regress (see section “Experimenter’s Regress”): to know if the instrument is reliable we need a true value; however, to get a true value we need a reliable instrument. And when measuring an opinion or an attitude we cannot forget whether there is a true value (see section “The Fidelity of Data and of Interpretations”).

For these reasons, evaluating the reliability of an instrument is a complex undertaking in social and organization sciences, just because the status of the object measured could be unstable: it may change between two observations, and do so even after a short time has elapsed. In other words, the statuses on certain properties may change both because of reasons external to the instrument (*natural* change) and because of the instrument itself (*induced* change). For example, an opinion or an attitude change as a consequence (induced) of the interview, because elicitation procedures make informants or interviewees conscious of themes or conditions of which they are not normally aware (Cicourel, 1988: 904). Hence, the observation process can modify the object (See the Heisenberg’s principle of indetermination) or the social actors’ behavior as the psychologist Elton Mayo and colleagues founded during their research at the Hawthorne plant of the Western Electric Company (called *Hawthorne effect*).

Hence, each method has specific problems of reliability, which are not equivalent and they must be dealt specifically. Such problems should be addressed with a “situational approach.” For example, in ethnographic research most of the issues about reliability risen by conventional methodology are not active or their dangerousness is limited. Problems of reliability certainly exist in participant observation^3^, but (from a practical perspective) they should not be exaggerated for several reasons. Firstly, because gathering is a long-drawn-out process involving numerous observations. Consequently, wrong information collected by the first observations can be corrected by subsequent ones. Secondly, unlike opinions, social practices (rituals, routines, and ceremonials) are stable processes (Gobo and Cellini, 2020: 123) unlikely to change during the time-span of a research project, unless it lasts for years (Burawoy, 2003), or unless the ethnographer is perceived as a spy or an informer (which infrequently happens). Thirdly, prolonged fieldwork (unlike a survey) allows elimination of the misunderstandings which certain questions may have produced, and which may have made the information imprecise. Finally, prolonged fieldwork also familiarizes the participants with ethnographic methodology. It thus reduces intrusiveness – although this is still the feature that most affects an instrument’s reliability.

In conclusion, in order to improve the replicability of their research findings, it is essential that researchers act upstream, improving data collection procedures. This can be done by checking the aspects detailed below.

### Appropriateness of Conceptualization

By means of conceptualization, the research establishes a relationship between the general concept and the indicators (or specific concepts).

**Strategies of nursing staff at a rest home**

After a week of observing the behavior of nursing staff at a rest home, the ethnographer decided that the strategy of keeping the elderly residents in bed as long as possible was a sign (a clue or an indicator) of the existence of practices designed to achieve greater social control. However, the next week, on talking with the manager of the rest home, and some nurses who had worked at the facility for some time, the ethnographer discovered that the practice had been introduced only recently, as an organizational response to a shortage of staff due to a cut in public funding.

Thus, the ethnographer first thought that the strategy was a suitable indicator for collecting information about a particular phenomenon (concept or category), but then realized that he had conceptualized it wrongly.

Posing the problem of the appropriateness of a conceptualization means enquiring as to whether the indicators considered are valid expressions of the concept in question. As Carmines and Zeller (1979: 101) point out, this is less a technical question (as in the case of operational definitions) than an eminently theoretical one.

However, assessing the appropriateness of an indicator is difficult. The conceptualization takes place ‘upstream’ from the operational definition, and therefore cannot be checked by the latter. Consequently, any errors in the conceptualization cannot be detected by the gathering procedures, because they lie ‘downstream’ from it. In other words, a systematic bias in the conceptualization due to a prejudice or a pre-assumption has a knock-on effect throughout a gathering’s progress. All theories run this risk.

Escaping from this circularity is difficult (and sometimes impossible), although the reflexive researchers, receptive to signals from the field and expecting the unexpected, are able to modify their conceptualization *as the research proceeds*. This reflexive process comes about (also) through the ‘documentary method of interpretation.’

### Trustworthiness of Operational Definition and Operational Acts

The operational definition concerns with the research design or plan. It refers to:

‘the whole set of rules driving operations by which the status of each case on the property X is noticed, assigned to one of the established category […] and recorded in the necessary way in order to start […] the analysis with the chosen techniques. Most of these rules are habits which generally rule some technical aspects of research […] other rules are more specific, and the researcher must to explicit them every time if s/he wants to transform the property X in a variable of his/her research’ (Marradi, 1980, p. 23)

Among those habits, conventions and rules, we have the lexical definition of index, the negotiation for the access to the field, contrivances (guarantees, informal contracts, tricks and so on) for overcoming the social actors’ distrust, the system of writing fieldnotes, control procedures about the validity of data. For example, coding is an activity driven by an operational definition. Observing and interpreting are ongoing implementation of several “operational act” (Schatzman and Strauss, 1973, p. 101). The ‘documentary method of interpretation’ proposed by Garfinkel is the qualitative version of the operational definition:

‘The method consists of treating an actual appearance as “the document of,” as “pointing to,” as “standing on behalf of” a presupposed underlying pattern. Not only is the underlying pattern derived from its individual documentary evidences, but the individual documentary evidences, in their turn, are interpreted on the basis of “what is known” about the underlying pattern. Each is used to elaborate the other’ (Garfinkel, 1967, p. 78).

The trustworthiness of operational definition and operational acts is noticed through a comparison between the *initial* research design (*a priori* trustworthiness) and the *final* outcome (*a posteriori* trustworthiness) of collecting data and analysis procedures. Retrospectively, the researcher may find that some cognitive and intellectual aims have been not achieved.

To deal with the problem of reliability, some social scientists have stated that clues related to the reliability of (i.e.) ethnographic observations can also be collected by means of *triangulation* procedures (Denzin, 1978; Jick, 1979; Fielding and Fielding, 1986). Taken from military strategy and navigation^4^, this term denotes in the social sciences the combined use of different methodologies in the analysis of a phenomenon. The data yielded by individual interviews, focus groups, questionnaires, or official statistics can be compared against the ethnographic data. Consistent results may confirm the reliability of the ethnography. However, if the results are partially consistent, or not at all, one may conclude that (a) the ethnography is unreliable; or (b) that the other methods are unreliable^5^; or (c) that the researcher has been not accurate.

### Researcher’s Accuracy

Qualitative research grants a great deal of autonomy to the researchers. They have broad discretion in choosing how to proceed, and when. However, it may not be certain that the observations reported (for example) by the ethnographer faithfully reflect the events observed. This doubt was raised, for example, by ethnomethodologists in regard to Goffman’s studies. The audience may therefore question the researchers’ accuracy and their ability to listen and understand. And there is also the possibility that accidental errors have been committed.

There are two distinct aspects to the problem of researchers’ accuracy: one ‘internal,’ concerning the researchers themselves; the other ‘external,’ regarding the audience and the scientific community.

### Internal Accuracy

The researchers can *improve* their accuracy by introducing demanding procedures into each phase of the inquiry. The research design and operational definitions, field access strategies, observation procedures, interview instructions, information collection techniques, principles of conversation transcription, the logic of data analysis, and write-up formats – all these provide the researcher with constant help and referents. Nonetheless, these constraints should not be regarded as obstacles to researcher’s smooth progress, for they are also (and above all) resources on which researchers – especially novices – can constantly draw.

### Accountability (or External Accuracy): Information for the Audience’s Evaluation of Research

Readers could see things differently. They want to know how to check the researcher’s accuracy. This introduces the problem of the ‘inspectionability’ of databases: in the sense that, as Alan Bryman points out, “field notes (…) are rarely available; these would be very helpful in order to allow the reader to formulate his or her hunches” (1988: 77).

A remedy to the problem is *accountability*, that is for the researchers to publish the empirical documentation on which their analysis has been based. They should therefore exhibit the main materials (observational notes, dialog transcripts^6^, photographs and drawings of the places observed) from which their conclusions have been drawn. Obviously, this can only be done to a partial extent, and only to support the most important findings, given the limited space made available for publications by book publishers and journals. However, in this way, ethnographers let themselves be evaluated by readers, and by the scientific community at large, on six main criteria (sketchily in section “Moving to a Situational and Organizational Approach”):

1. *completeness*: descriptions must be accompanied by details of the context;
2. *saturation* of the categories: all events must be covered by the concepts proposed, with cognitive dissonances eliminated and the marginal utility of limit cases evaluated;
3. *authenticity*: the fieldwork must be certified as genuine;
4. *consistency*: the extent to which events observed on different occasions are allocated to the same category;
5. *credibility*: the consistency between descriptions and interpretations. The following requirements should be fulfilled: the results are consistent with the theory adopted; the concepts have been systematically correlated; the causal relations have been developed correctly;
6. *plausibility*: consistency between the researchers’ conclusions and the consolidated knowledge of their scientific community. This criterion should not be too rigorously applied, however, for otherwise the conclusions will merely replicate the disciplinary *status quo*.

According to the sociologist Randall Collins, this last criterion is the most important of all:

‘the most important way in which the validity of a theory can be established is by showing the coherence of its explanatory principles with other well-grounded theory (…) Qualitative microsociology, for example, or observation-based organizational studies, have not depended upon statistics. Their validity and their contributions to our knowledge — which I would say have been considerable — come from their degree of coherence with all our accumulated theoretical principles’ (Collins, 1988: 504–505).

If a team is doing the research, the accuracy issue assumes different features: the presence of several researchers may be a problem but it is also an important resource. Although the problem of consistency arises in the allocation of events to the same category (Hammersley, 1992: 67), accidental errors and distortions can be corrected or reduced by comparisons among the researchers. Consistency among codes can be further increased if they are generated by CAQDAS softwares. These provide greater transparency and facilitate intersubjective agreement among the researchers, thereby ensuring greater accuracy.

## The Observational Relation

Another aspect (often overlooked), which contributes to the replicability of the research findings, is the type of observational relation established by the researcher with the participants. It is, in part, beyond the researcher’s control and consists of: (a) the goffmanian “face” that the participants want to convey of themselves; (b) the organizational constraints on the researcher’s work; (c) the extent to which the participants wish to be observed and so on.

After the researchers have attempted to manage positively these aspects and remedy any difficulties that may arise during the research, the only option is to give the audience information (‘*impressionist tales*’ as John Van Maanen calls them) about this observational relation so that they have further material for evaluation of the contingency of the data. This information may be about: the conditions in which the research has been conducted; the constraints on the researcher which have restricted the observational field; the helps and hindrances encountered, and which of the participants were responsible for them; the requests and permissions granted or refused; problems of adapting to the field; the particular interests emerging from interviews, from correspondence, from conversations or telephone calls. Cicourel (1968) recalls that it was only after he had published the results of his research on juvenile courts that he learned that the police-officer participants (notwithstanding their apparent frankness) had been invariably reticent. He recounts that after the police officers had read his book, they commented to him roughly as follows: “if we’d known that this was the purpose of your research, we’d have told you a whole lot of other things…”.

Publication of this information is a sort of ‘natural’ history of research, to which one devotes a chapter of a report, as did Whyte (1955) with the *Appendix*, Cicourel (1974) with the chapter *Notes on The Argentine Historical Context and Some Ethnographic Impressions of Buenos Aires* or Mehan et al. (1986) with the chapter titled *The ethnographic context of the study*. This natural history, like the aspects treated in the previous sections, does not guarantee the authenticity of research descriptions, but it does give the audience, and more generally the scientific community, good grounds for deciding whether to accept or to reject these descriptions. Also pertaining to a natural history of research are, according to Strauss and Corbin (1990: 253): discussions of the criteria on which the sample has been selected; how and when both the categories and the main hypotheses were formulated; for what reasons other hypotheses were discarded. Finally, it is also important to specify the procedures followed in confirming the hypotheses.

## The Fidelity of Data and of Interpretations

After examining in detail various aspects that are generally neglected in the conventional concept of reliability, the time has come to address the other cornerstone of replicability: validity.

Let us suppose that the conceptualization (i.e., the indication relationship) has been appropriate; that the operationalization (i.e., the move from the indicators to the relative variables) has been correct; that the various techniques have been reliable; that the researchers have been precise and accurate; and, finally, that the observational relation has been successful. Can we be sure that they have correctly interpreted the situations observed? No, we cannot, because two forms of imprecision can always occur:

1. at the *data* level: a discrepancy between the state recorded and the actual status (Marradi, 1990: 82);
2. at the *interpretation* level: a discrepancy between the researcher’s interpretations and the social phenomenon they refer to Hammersley (1990: 57).

The assumptions underlying these two statements introduce a specifically epistemological issue. It concerns the conventional ‘truth theory,’ grounded on the correspondence between observation and reality, and which assumes that:

‘there is the real world of people, events and circumstances, on the one hand, and one’s own observations and descriptions of this world, on the other hand. Competent observation and description depend principally on achieving certain formal relationships between the former and the latter – that is, by producing good ‘pictures’ of reality’ (Schwartz and Jacobs, 1979: 256).

This realist theory of truth, which has bred the concept of ‘validity,’ presupposes that an effectively knowable status exists: an assumption, which the postmodernists have assailed for decades. Yet still absent from the debate is treatment of this important issue from an organizational standpoint, case by case, and eschewing conflict waged solely on principles and partisanships. It is therefore necessary to abandon the concept of validity and replace it with two epistemologically more appropriate concepts: fidelity of data and fidelity of interpretations.

### Validity Re-framed

Firstly, *organizationally*, there are some situations where ‘facts,’ or an objective reality, exist; and there are some situations where ‘facts’ do not exist. In other words, there are different levels of ‘facticity.’ Hence, in the social and organizational sciences, adopting a truth theory wholly based on correspondence is problematic, because it can only apply to a *few* (albeit important) individual properties of the social world, generally constructed by bureaucratic and organizational processes (See Marradi, 1990: 81): demographic attributes (such as nationality, place of birth and residence, educational degree and so on) and in general all those such as (for example) having a driving license, a criminal record, a fine or being on the electoral register. The distinctive feature of these attributes is that the official record (produced by an organization) not only certifies their status but overall *constitutes* it^7^.

Although official records are certainly artifacts that someone has constructed, however, once they have been constructed, they become… real. In fact, if the status of citizenship is attributed to an individual by mistake, s/he (even without having the requirements) will be entirely citizen of that specific country, because the record constitutes such status.

Hence, a realist theory of truth may be appropriate for certain attributes, whose it is possible to check whether the respondent is lying or not about them: for example, we cannot say (subjectively) that we are doctors if there nowhere exists a record, which (objectively) constitutes this status of ours. In fact, if the record that states we are doctors has been lost or destroyed (e.g., because of a war in our country), we are not doctors anymore.

Unlike, a realist theory of truth does not apply to the majority of the properties with which researchers concern themselves, because “the actual statuses on some continuous property are not knowable to us, and discrete properties are not measurable in the strict sense of the term” (Marradi, 1990: 82).

In other words, attitudes, motivations, opinions do not have effective statuses.

### Fidelity as Successful Prediction

To evaluate the fidelity of a statement, one possible criterion is its degree of success in predicting. As the philosopher of science Mary Hesse (1988) argues, in her polemic against radical constructivists, the fact that a certain number of fatal diseases have been reduced and controlled through identification of something (a virus, for example), which can be paraded, demonstrates the capacity of human beings to conform with an external reality. Such success proves this adjustment. More generally, survival testifies to a success. It is proof that not all cognitive inductions and procedures are purely arbitrary or completely conventional.

Likewise, an ability to imitate the practices of participants to the point that one is regarded for all practical purposes as a competent member of the community (like the anthropologist Richard K. Nelson, who learned how to hunt like the Eskimos) may be indicative of the fidelity of a researcher’s statements. This also seems to be Goffman’s opinion in regard to the study of mental illness:

‘as Harold Garfinkel has suggested, we should be in a position (not desirable in itself but desirable as a test of theory) to program insanity, that is, reduce to a minimum the instructions you would have to give an experimental subject in order to enable him beautifully to act crazy’ (Goffman, 1964: 140).

There are two obvious objections to this position. The first has been raised by Geertz and the ethnomethodologists: it is never possible to furnish complete instructions, precisely because instructions are intrinsically vague and indeterminate (Mehan and Wood, 1975: 233–5; Schwartz and Jacobs, 1979: 258). The second is that research situations do not always permit this attempt. Nevertheless, it is still extremely useful, when possible, to make small-scale predictions and observe their results. The fulfillment of certain predictions or the confirmation of a hypothesis may increase the fidelity of the researchers’ statements and heighten intersubjectivity with their audience. However, they cannot guarantee certainty, because the success may be due to intervening variables unknown to the researcher.

### Participant Validation

A further criterion that reflexive methodology ought to embrace in evaluating the fidelity of data or an interpretation (or a researcher’s account) is to obtain confirmation or denial (verbal or written) from the participants. This procedure has been variously called *member verification*, *host verification*, *member test of validity*, *respondent validation*, *group feedback analysis*, *member validation*. For this purpose, the researcher conducts individual interviews or organizes discussion groups with the actors. The procedure requires that the researcher’s description or theory be expounded simply and clearly, in conformity with Schutz (1953: 45) ‘postulate of adequacy,’ so that the scientific models of social action are understandable to the participants. This does not mean that there should also be higher-order concepts, because researchers and actors have different jargons and different stocks of knowledge.

Participants may be requested to validate both descriptive statements (the recorded data or status) and interpretative ones (an explanation, a hypothesis or a theory). These are two different epistemological problems. In fact, in interpretative statements, denial by the participants may trigger long and tortuous negotiations on the fidelity of the researchers’ interpretations.

**Member validation in a psychiatric organization**

The American ethnographers Robert M. Emerson and Pollner (1988) conducted research on the management of psychiatric emergencies in a regional Community Mental Health Clinic in southern California. Over a period of 6 months, they observed the work of psychiatric emergency teams (PET), these being mobile units consisting of psychiatric personnel deployed in the community in response to calls.

About 1 year after conclusion of their field research, Emerson and Pollner had completed the drafts of two papers on PET. These they presented to an assembly of 35 people, of whom about ten belonged to the regional clinic. Their purpose was to elicit feedback from members of a directly observed team. Contrary to Emerson and Pollner’s expectation of receiving clear feedback, their attempt at member validation obtained very ambiguous results. The recording of the event enabled Emerson and Pollner to reconstruct the obstacles against transparent feedback in detail:

• the participants could not fully understand the two papers because they were unfamiliar with the researchers’ language;
• they could not recognize themselves in the researchers’ descriptions and explanations because they were unaware of the motives for, and consequences of, their behavior;
• they considered the two papers to be not only ‘scientific’ but also ‘political,’ and thought that their critical findings threatened the existence of their organization;
• some participants agreed with the researchers’ accounts; others partially or totally disagreed with them;
• the participants’ responses were ambiguous, in the sense that they did not reject, but nor did they accept, the researchers’ accounts; hence it was unclear whether they could be taken as confirming or confounding the researchers’ interpretations.

Emerson and Pollner concluded that it was entirely natural that evaluation of a research report should mix interests of various kinds (scientific, political, personal, etc.), because validation never takes place in a vacuum, but always in a specific social and organizational context. However, they were still optimistic about the technique’s usefulness:

‘although these [verification] transactions are (…) problematic (..) it is because validation episodes often comprise intense moments of organizational and interactional life that they are capable of revealing aspects of the setting or organization in a new light’ (1988: 189).

Owing to the reflexive property of action, ethnographic accounts do not simply describe things; they also ‘execute moral evaluations, produce political, moral, and social consequences, and so on. Description are almost always ‘doing’ many more things in the social situation than simply ‘reporting’ a set of facts’ (Schwartz and Jacobs, 1979: 51).

Indeed, Bloor (1983) and Fielding and Fielding (1986: 43) argue that member validation is only a further source of information. Although it yields valuable *extra* information which enriches the empirical documentation collected by the researcher, it is not a means with which to evaluate the scientific validity of a research account.

The reason for the likely incomprehension between researcher and participants is practical, but it is also gnoseological: the incommensurability between the perspectives of the observer and the observed. As Schutz writes:

‘The meaning of an action is necessarily a different one (a) for the actor; (b) for his partner involved with him in interaction (…) and (c) for the observer not involved in such relationship (…) The constructs of the observer are, therefore, different ones than those used by the participants in the interaction, if for no other reason than the fact that the purpose of the observer is different from that of the interactors and therewith the systems of relevancies attached to such purposes are also different’ (1953: 24 and 26–27).

Given this impossibility, researchers need ‘only’ concern themselves with correctly grasping the common-sense meaning that action has for participants; they should not persuade them of that meaning. As the anthropologist Moerman (1974: 68) has stressed, social scientists should describe and analyze the ways in which concepts are used by the participants, and not simply – as the natives do – use them as explanations. Although scientific explanations should not disregard the meanings expressed by actors, they lie at a different level from those meanings. The participants may accept or reject them, but this should not necessarily affect the researcher’s interpretative statements. As Fielding and Fielding put it: ‘there is no reason to assume that members have privileged status as commentators on their actions (…) such feedback cannot be taken as direct validation or refutation of the observer’s inferences’ (1986: 43).

## Concluding Remarks: Between Normativism and Postmodernism

In the past, feminist and postmodern approaches have rightly diagnosed the ‘legitimation crisis’ (Denzin and Lincoln, 2000: 17), meaning by this expression the qualitative research cannot be evaluated with conventional criteria. However, the solution they proposed (abandoning any reference to the credibility of qualitative research, substituting moral values and political positions as guarantors of standards – See Seale, 2004: 409) avoids facing the epistemological core of the problem of replicability. Unlike, the ‘legitimation crisis’ can be re-thought and tackled introducing new “politics and poetics of legitimation,” based on a different conceptualization of the whole validation process, where replicability is an important tile. They pertain the attention to aspects neglected by the conventional concepts of reliability and validity, the re-frame of the latter in an organizational, practical and constructivist epistemological framework, and a conscious use of the argumentative and rhetorical strategies (See Gobo, 2008, chap. 15) in order to validate the research findings.

Calling for a ‘practical and linguistic turn’ on issues concerning the legitimation of qualitative research does not entail abandoning every prescriptive claim, that is, renouncing all research conventions on how to inquire. Between the normative (rule-based) approach of conventional methodology and the libertarian (rule-free) attitude of postmodern radicalism or methodological anarchism, the “situational approach” argues that methodological conventions can indeed be constructed, but they must derive from careful observation of (and reflection on) the research practices and the nature of that particular research method (e.g., in depth interview, observation, tape or video recording, focus group) selected by the researcher.

Because research methods are not just (neutral or interchangeable) tools, but they have an inner force (as the language in the Austin’s speech act theory). There is a performativity effect of each method, which has (according to the actor-network theory by Michel Callon, Bruno Latour and John Law – see Law, 1986) an agency. Each method incorporates a specific epistemology, defined methodological worldview. It embodies a distinct capacity of (partially) constructing data. Hence, methods highly concur (with the researcher, the participants, the research setting, the organizational and institutional constraints and opportunities) to build the data (Gobo, 2016). This is why data collected by survey interviews, discursive interviews, focus groups, ethnographies and so on, are often different, never overlap and not rarely conflict. Because there is a strong link (though not deterministic, of course) between the type of datum collected and the type of research method: what you get with a certain method, you do not catch with another one (See Becker and Geer, 1960, for a comparison between participant observation and “conversational interview”). Methods are like fruit trees: each tree produces a specific fruit. For these reasons, researchers should ask themselves: is the method I chose intrusive or not adequate to the situation under study? If we want to study collective behaviors, is discursive (in-depth) interview an adequate technique or ethnography has to be preferred? What is the best method to study attitudes?

Still too often, both the normative and postmodernist approaches address methodological issues solely at the level of abstract principles. A third way envisions the chance of combining a loosely normative endeavor, a “methodological situationism” and a reflexively grounded epistemology.

For example, it is often argued that ethnography is a *highly* subjective method, in the sense that it is susceptible to the influences of the researcher’s attitudes and perceptions. In other words, if different researchers visit the same setting, they will see different things, and their ethnographic notes will record different aspects. Instead, a questionnaire or an in-depth interview, if conducted correctly, is more likely to obtain similar replies (ensuring reliability) regardless of who the interviewer is. There is, however, scant empirical evidence for this assertion. What makes ethnographic findings reproducible and replicable is the fact that it observes behaviors (rituals, routines, ceremonies), which are considerably more stable over time than attitudes and opinions (Gobo and Molle, 2017). Those who work with organizations know very well that more time is required to alter a behavior than an attitude, not to mention opinions, which can be so volatile that they change from 1 day to the next.

From this, it follows that, because behaviors are temporally rather stable, the results of ethnographic research can be repeated and reproduced. This depends on two factors: the presence of a precise research design to guide the research, and that no significant organizational or institutional changes take place between one study and the next. For this reason, ethnographic finding could be (in certain situations) generalizable.

If we abandon the positivistic idea that an objective reality exists independently of the observer, the problem of the correctness and veracity of the researcher’s statements shifts to a broader dimension, where it is not so much the truth (which, as we saw, is often impossible to ascertain) that matters as the researchers’ capacity to persuade the audience of the credibility of their statements conclusion.

## Data Availability Statement

The raw data supporting the conclusions of this article will be made available by the authors, without undue reservation.

## Author Contributions

The author confirms being the sole contributor of this work and has approved it for publication.

## Conflict of Interest

The author declares that the research was conducted in the absence of any commercial or financial relationships that could be construed as a potential conflict of interest.

## Footnotes

1 In qualitative research the term ‘reliability’ has been introduced also by Hammersley as “the degree of consistency with which instances are assigned to the same category by different observers or by the same observer on different occasions” (1992, p. 67). However, the Hammersley’s definition does not refer to the conventional reliability but to the observers’ *accuracy*. In fact, he puts the method on the same level as the researcher who uses it. However, they are two different entities that should not be confused; also because subjectivity is a property of the latter only. In addition, collecting data and analyzing them are quite different processes. Hammersley’s definition might fit better with the second process.

2 In social science, the possibility of lying is widely acknowledged to the “object” of one’s research. In the physical and natural sciences, the same agency is not acknowledged (at least for the moment…) to atoms, cells, etc. Even more reason to stop trying to emulate natural sciences.

3 In non-participant observation, by contrast, this risk does not exist because the observer is not noticed by the participants.

4 In navigation, three fixes are usually made to determine a nautical position.

5 Hammersley and Atkinson (1983: 199), Silverman (1993: 156–8; 2000: 99), Mason (1996: 27), are highly critical of triangulation, doubting the methodological soundness of comparing data collected with different instruments and cognitive intentions.

6 This is a common standard in discursive and conversational analysis studies.

7 This implies that the postmodern theories that oppose a realist theory of truth should temper the absolutism of their assertions.
